# An Ethanolic Extract of *Cucurbita pepo* L. Seeds Modifies Neuroendocrine Disruption in Chronic Stressed Rats and Adrenal Expression of Inflammatory Markers and HSP70

**DOI:** 10.3389/fphar.2021.749766

**Published:** 2021-11-10

**Authors:** Hailah M. Almohaimeed, Shereen Hamed, Hanan S. Seleem, Ashwaq H. Batawi, Zuhair M. Mohammedsaleh, Maha Jameal Balgoon, Soad S. Ali, Soad Al Jaouni, Nasra Ayuob

**Affiliations:** ^1^ Department of Basic Science, College of Medicine, Princess Nourah Bint Abdulrahman University (PNU), Riyadh, Saudi Arabia; ^2^ Department of Medical Histology and Cell Biology, Faculty of Medicine, Mansoura University, Mansoura, Egypt; ^3^ Histology Department, Faculty of Medicine, Menoufia University, Shebin ElKoum, Egypt; ^4^ Department of Basic Medical Sciences, Unaizah College of Medicine and Medical Sciences, Qassim University, Buraydah, Saudi Arabia; ^5^ Department of Biological Science, Faculty of Science, King Abdulaziz University, Jeddah, Saudi Arabia; ^6^ Department of Medical Laboratory Technology, Faculty of Applied Medical Sciences, University of Tabuk, Tabuk, Saudi Arabia; ^7^ Department of Biochemistry, Faculty of Science, King Abdulaziz University, Jeddah, Saudi Arabia; ^8^ Department of Histology and Cell Biology, Faculty of Medicine, Assuit University, Asyut, Egypt; ^9^ Yousef Abdullatif Jameel Chair of Prophetic Medical Applications (YAJCPMA), Faculty of Medicine, King Abdulaziz University, Jeddah, Saudi Arabia; ^10^ Department of Hematology/Pediatric Oncology, King Abdulaziz University Hospital (KAUH), Faculty of Medicine, King Abdulaziz University, Jeddah, Saudi Arabia; ^11^ Department of Medical Histology, Faculty of Medicine, Damietta University, Damietta, Egypt

**Keywords:** pumpkin, stress, depression, caspase-3, ki67, Hsp70, apoptosis

## Abstract

**Background:** Pumpkins (*Cucurbita pepo* L.) were described to have antioxidant, anti-inflammatory, anti-fatigue, and antidepressant-like effect. The adrenal gland is an important stress-responsive organ that maintains homeostasis during stress.

**Objectives:** This study aimed to assess the efficacy of the administration of *Cucurbita pepo* L. (CP) extract in relieving behavioral, biochemical, and structural changes in the adrenal gland induced by exposure to chronic unpredictable mild stress (CUMS) and to explore the mechanism behind this impact.

**Materials and Methods:** Forty male albino rats were divided into 4 groups (*n* = 10): control, CUMS, fluoxetine-treated, and CP-treated groups. Behavioral changes, corticosterone level, pro-inflammatory cytokines TNF-α and IL-6, and oxidant/antioxidant profile were assessed in the serum at the end of the experiment. Adrenal glands were processed for histopathological and immunohistochemical assessment. Gene expression of caspase-3 and Ki67 and heat shock protein 70 (HSP70) were assessed in adrenal glands using RT-PCR.

**Results:** The CP extract significantly reduced the corticosterone level (*p* < 0.001), immobility time (*p* < 0.001), and inflammatory and oxidative changes associated with CUMS-induced depression compared to the untreated group. The CP extract alleviated CUMS-induced adrenal histopathological changes and significantly reduced apoptosis (*p* < 0.001) and significantly upregulated antioxidant levels in the serum.

**Conclusion:** *Cucurbita pepo* L. effectively ameliorated the chronic stress-induced behavioral, biochemical, and adrenal structural changes mostly through its antioxidant and anti-inflammatory effects.

## Introduction

More than 264 million people of all ages suffer depression all over the world (WHO, 2020). The performance of the depressed person is generally poor at school, at work, and in the family. Depression may lead to suicide; so, it is considered the second leading cause of death in 15- to 29-year-olds (WHO, 2020). Mammals can survive stressful events by activation of appropriate physiological responses to these events. The adrenal gland is part of the hypothalamic–pituitary–adrenocortical (HPA) axis and the sympatho–adrenomedullary axis that maintain homeostasis during stress (Ulrich-Lai et al., 2006). It was reported that exposure to thermal stress results in rapid expression of HSP70 in the adrenal cortex (Huang et al., 2001). These proteins share intracellular trafficking, antigen presentation, apoptosis, and many other actions (Khar et al., 2001). It has been described that exogenous hormones or interference with endogenous hormones, during the critical periods of development, can have permanent effects on the physiological and behavioral pathways regulated by hypothalamic neuroendocrine circuits (Gore and Patisaul, 2010). Neuroendocrine disruption was also described to extend the concept of endocrine disruption to include the full breadth of integrative physiology; therefore, it is more than hormonal upset (Waye and Trudeau, 2011).

Changes in neuroendocrine regulation, metabolism, and diet/microbiota are considered triggers for inflammation and predispose to developing depression (Huang et al., 2019). It was postulated that depression—one of the neuropsychiatric disorders—and inflammation have a two-way relationship. While depression promotes inflammatory reactions, inflammation promotes neuropsychiatric disorders including depression (Bauer and Teixeira, 2019).

Fluoxetine, a classical antidepressant, is among the drugs available in the market for treatment of depression. It is now considered as an emerging neuroendocrine disruptor. This effect of fluoxetine is a side effect, rather than main therapeutic targets in mammals (León-Olea et al., 2014). Therefore, there is a need for safer antidepressant with no side effects on the neuroendocrine status.

Pumpkins (*Cucurbita pepo* L.) are economically important species cultivated worldwide. They have nutritional and health benefits as they are rich in phenolics, flavonoids, amino acids, carbohydrates, and vitamins (Wang et al., 2002). Several research studies revealed that pumpkins have extensive bioactivities, such as antidiabetic, anticancer, antioxidant, anti-inflammatory, anti-fatigue, and antidepressant-like effect (Wang et al., 2012; Zhang et al., 2012; Nawirska-Olszańska et al., 2013; Kim Nr et al., 2016). Traditional medicine mainly Ayurvedic systems and Chinese medicine have used different parts of the pumpkin, including flesh of the fruits and seeds (Perez Gutierrez, 2016). The antidepressant efficacy of Sweetme Sweet Pumpkin (SSP) and *Cucurbita moschata Duch* was previously tested in an *in vivo* study using a forced swimming test (FST)–induced animal model of depression and was compared with fluoxetine (Kim Nr et al., 2016). In one of the relatively recent reviews that summarized that the antidepressant foods, such as pumpkin seeds, were described to have an antidepressant food score of 47%, providing its antidepressant-like effect (Lachance and Ramsey, 2018). Recently, Dotto and Chacha endorsed conducting more animal- and clinical trial-based research studies in order to confirm the ameliorative effect of pumpkin seeds on depression (Dotto and Chacha, 2020).

These reports were encouraging to test the efficacy of pumpkins in alleviating the impact of chronic stress on the adrenal glands. Therefore, this study was performed to assess the effect of oral administration of the *Cucurbita pepo L.* (CP) extract on relieving the chronic unpredictable mild stress (CUMS)–induced behavioral, biochemical, and adrenal structure and to explore the mechanism behind this impact.

## Materials and Methods

### Extraction and Dosage of Pumpkin

*Cucurbita pepo L.* (voucher specimen: AQJ_95) were purchased from the local market at Jeddah, Saudi Arabia. They were identified in the King Abdulaziz University herbarium using specimens of herbarium and the flora of KSA (Chaudhary, 2001). Voucher specimens were deposited in the herbarium. CP was identified by the authors and was verified by a botanist from the Faculty of Science, King Abdulaziz University.

Extraction of CP was done according to the previous studies (Wang et al., 2012)*.* The raw fruits with the peel were cut using a slicer and dried using a lyophilizer (FD5508; ilShinBioBase Co., Ltd., Korea) and then crushed by using an electrical grinding machine. The powder was passed through a 40-mesh sieve to get a fine powder and stored in an airtight container. The dried powder (50 g) was mixed with 450 ml of 80% ethanol and left for 1 day at 37°C in a shaker (JSSI-100T; JS Research Inc., Compact Shaking Incubator., Korea) and then was filtered with cotton and filter paper on the second day. This extraction process was repeated twice at 37°C to get an ethanol extract.

### Identification of the Constituents of CP

The chemical composition of PE was analyzed using a trace gas chromatography GC-TSQ evo 8000 mass spectrometer (Thermo Scientific, Austin, TX, United States) with a direct capillary column TG–5MS (30 m × 0.25 mm × 0.25 µm film thickness). The column oven temperature was initially set to 50°C and then increased by 5°C/min to 250°C and held for 2 min and then increased to a final temperature of 300°C by 25°C/min and held for 2 min. The injector and MS transfer line temperatures were kept at 270 and 260°C, respectively; helium was used as a carrier gas at a constant flow rate of 1 ml/min. The solvent delay was 4 min, and diluted samples of 3 µl were injected automatically using an Autosampler AS1300 coupled with GC in the splitless mode in a PTV injector. EI mass spectra were collected at 70 eV ionization voltages over the range of m/z 50–650 in the full scan mode. The ion source temperature was set at 250°C. The components were identified by comparison of their mass spectra with those of WILEY 09 and NIST 14 mass spectral database that are used to identify and study the chemical composition of unknown components in any extract (Wiley, 2006; Mikaia et al., 2014). Analysis had been done in qualitative type using Thermo Scientific™ Xcalibur™ 2.2 software, and all values were reported in relative percentage (Abd El-Kareem et al., 2016).

### Experimental Design

This study was approved by the Biomedical Research Ethics Committee at the Faculty of Medicine, King Abdulaziz University, Jeddah, KSA (reference number 45-20). In this study, forty male albino rats weighing 150–200 g and aged 2–3 months were obtained from the King Fahd Medical Research Center (KFMRC). Before starting the experiment, the rats were left to acclimatize to the laboratory conditions for 1 week. Ten rats were assigned as the control group which was left unexposed to CUMS. The other thirty rats were subjected to the procedure of CUMS for 4 weeks that included different types of stressors at different times during the day in order to prevent habituation to stress. The CUMS procedure was fully described in previous works (Ayuob et al., 2016; Ali et al., 2017) and was shown in Table 1. The rats exposed to CUMS were divided into 3 groups (*n* = 10). The untreated group (CUMS) received the vehicle 0.03% carboxymethyl cellulose (CMC-Na) by gavage for 2 weeks. The FLU-treated group received FLU (Dar Al Dawa Pharmaceuticals Co., Ltd., Amman, Jordan), an antidepressant used for pharmacological validation, dissolved in CMC-Na 0.03% at a dose of 20 mg/kg by gastric gavage (Li et al., 2014). The CP-treated group received the CP extract dissolved in distilled water at a dose of 100 mg/kg by gavage for 2 weeks according to Wang et al. (2012).

**TABLE 1:** List of the stressors used in the CUMS protocol used in this study during the 1st week. These stressors were repeated during the 2nd, 3rd, and 4th weeks at different time points (Doron et al., 2014).

	Week
Day 1	Restraint stress (4 h)
Day 2	Placement in an empty cage with water at the bottom and lights on (4 h)
Day 3	Title cages at 30° (4 h)
Day 4	Placing mice in cages with wet sawdust (4 h)
Day 5	Placing mice in soiled cages of other mice (4 h)
Day 6	Restraint stress (4 h)
Day 7	Reversal of the light/dark cycle (4 h)

### Behavioral Changes Assessment

The FST was performed after 4 weeks to confirm the effect of CUMS on the rats (Ali et al., 2017). During this test, each rat was placed in a glassy cylindrical container (height 20 cm, diameter 14 cm) with 15 cm of water at 25 ± 2°C. The rat was videotaped for 6 min using behavior software (Noldus Information Technology, EthoVision XT®), and the total time spent immobile during the 6 min was measured by a technician blind to the experiment groups. The total time, in seconds, spent by the rats without limb movement, except for the minor movement necessary to keep the mouse afloat, “immobility time” during the 6 min was determined.

### Biochemical Techniques

Twenty four hours after finishing the behavior test, blood samples were taken from the intra-orbital sinus of rats after being anesthetized with 4% isoflurane (SEDICO Pharmaceuticals Company, Cairo, Egypt) in 100% oxygen. After that, the rats were euthanized by cervical dislocation. Blood samples were centrifuged at 3,000 rpm for 15 min at 4°C to obtain the serum and were kept at −18°C for biochemical assessment.

The level of corticosterone (ALPCO Diagnostics, Orangeburg, NY, United States) was assessed using enzyme-linked immunosorbent assay kits according to the manufacturer’s instructions. According to the manufacturer’s instructions, TNF-α and IL-6 (Quantakin R&D system, USA Kit) were assessed by using enzyme-linked immunosorbent assay. The optical density of each sample was determined in duplicate with a microplate ELISA reader set to 450 nm.

The thiobarbituric acid reactive substances (TBARS) assay kit (Biodiagnostic; Egypt) was utilized to measure the level of malondialdehyde (MDA) spectrophotometrically at 535 nm (Gamal et al., 2018). The level of superoxide dismutase (SOD) was measured by using the SOD assay kit (Biodiagnostic; Egypt) (Packer, 2002). The assessment of the glutathione peroxidase (GPX) level was performed by using the GPX kit (Randox Labs, Crumlin, United Kingdom). To quantify the activity of catalase (CAT), a calibration curve was generated for the assay and all samples, using assay kits (Biodiagnostic; Egypt). The method is previously described (Gamal et al., 2018).

### Quantitative Real-Time PCR

RNA extraction from the tissue samples was done using the TriFast™ reagent (PeqLab, Germany, Cat No.: 30-2010), as described in the manufacturer’s protocol. The concentration of the purified RNA was estimated by using a NanoDrop 2000c spectrophotometer (Thermo Scientific, United States). The extracted RNA from each sample was reverse transcribed using the SensiFAST™ cDNA synthesis kit for RT-qPCR (Bioline USA Inc., United States, Cat No.: BIO-65053), following the manufacturer’s instruction. The synthesized cDNA was stored at -80°C until utilization for qRT-PCR.

The qRT-PCR reactions were performed using the SensiFAST™ SYBR Lo-ROX kit (Bioline USA Inc., United States, Cat No.: BIO-94002) on the Applied Biosystems 7500 real-time PCR detection system (Life technology, United States). Gene-specific primers for rats, used in this study, were designed by Primer3 software (v.0.4.0),and their specificity was checked using NCBI/Primer-BLAST program. The primers were then purchased from Willowfort™ (United Kingdom). The primers were GAPDH (5′-TGC​ACC​ACC​AAC​TGC​TTA​GC-3′, 5′-GGC​ATG​GAC​TGT​GGT​CAT​GAG-3′), caspase-3 (forward 5- TGT​ATG​CTT​ACT​CTA​CCG​CAC​CCG-3, reverse 5-GCG​CAA​AGT​GAC​TGG​ATG​AAC​C-3), HSP70 (5′-ACG​AGG​GTC​TCA​AGG​GCA​AG-3′, 5′-CTC​TTT​CTC​AGC​CAG​CGT​GTT​AG-3′).

Ki67 (forward 5-AGA​AGA​GCC​CAC​AGC​ACA​GAG​AA-3, reverse 5-AGA​AGA​GCC​CAC​AGC​ACA​GAG​AA 3). The PCR mixture was prepared as follows: 10 µl SensiFAST^TM^ SYBR Lo-ROX mix, 0.8 µl forward primer, 0.8 µl reverse primer, 2 µl template cDNA, and 6.4 µl nuclease-free water. The reaction mix was transferred to a thermal cycler that was previously programmed to hold at 95°C for 2 min, followed by 40 cycles of 95°C for 15 s, and then 60°C for 30 s. A negative control reaction containing no template was run in each experiment.

Melting curve analysis was carried out to prove specificity of PCR products, and the Ct value for each reaction was obtained from amplification plots. The relative quantification for each gene expression in the tissue samples was calculated using the comparative threshold (ΔΔCt) method with GAPDH as the internal control gene. For the overall fold change, it was calculated and linearized by the 2^−ΔΔCt^ arithmetic formula.

### Histological Techniques

At the end of the experiment and after the rats were anesthetized, the abdomen was opened and the right adrenal gland was dissected out. To obtain paraffin blocks, fixation of the adrenal gland in 10% neutral buffered formalin and further processes were performed. The paraffin sections of 4-μm thickness were prepared and stained with hematoxylin and eosin (H&E). Moreover, other paraffin sections were immunohistochemically stained using the streptavidin–biotin–peroxidase technique. Slides were incubated overnight at 4°C, and then they were incubated with monoclonal anti-Ki67 (Dako Cytomation, United States, at dilution 1:1,000) for demonstration of cell proliferation. In addition, polyclonal anti-caspase-3 antibody (Santa Cruz Biotechnology, United States, at dilution 1:1,000) was utilized for the detection of apoptosis. A polyclonal antibody against heat shock protein-70 (HSP70) (Dako, Carpinteria, CA, United States, at dilution 1:1,000) was also utilized. Corresponding biotinylated conjugated secondary antibody from the Dako staining system was used. The slides stained with the secondary antibody only were used as negative controls.

The nuclei were counterstained with hematoxylin. Brown cytoplasmic staining was considered a positive reaction. The stained sections were examined and photographed using an Olympus Microscope BX-51 (Olympus) connected to a digital camera and a computer. Semiquantitative analysis of antibody immunoreactivity was done by Pro Plus image analysis software. The percentage area of the immunopositive reaction was assessed in 30 fields at ×400 magnification. The positive cells were counted per 1.0 mm^2^ of area, as described by Zhou et al. (2016). At least five fields from each slide were examined, and the mean was calculated for each animal.

For morphometric analysis, four sections were examined in each animal (magnification ×100). The thickness of different zones of the adrenal gland was measured in micrometers. The adrenal cortex thickness was obtained by measuring the distance between the medulla and the adrenal capsule in a straight line, one measurement being taken in each quadrant of the adrenal cortex.

### Statistical Analysis

The behavioral, biochemical, and immunohistochemical data were analyzed by the Statistical Package for the Social Sciences (SPSS) version 16. The study variables were affected by two independent factors: stress exposure and treatment. Therefore, analysis using a mixed-model two-way ANOVA based on Bonferroni post hoc was performed. Statistical significance was considered at *p* < 0.05.

## Results

### Behavioral Results

**FIGURE 1:**
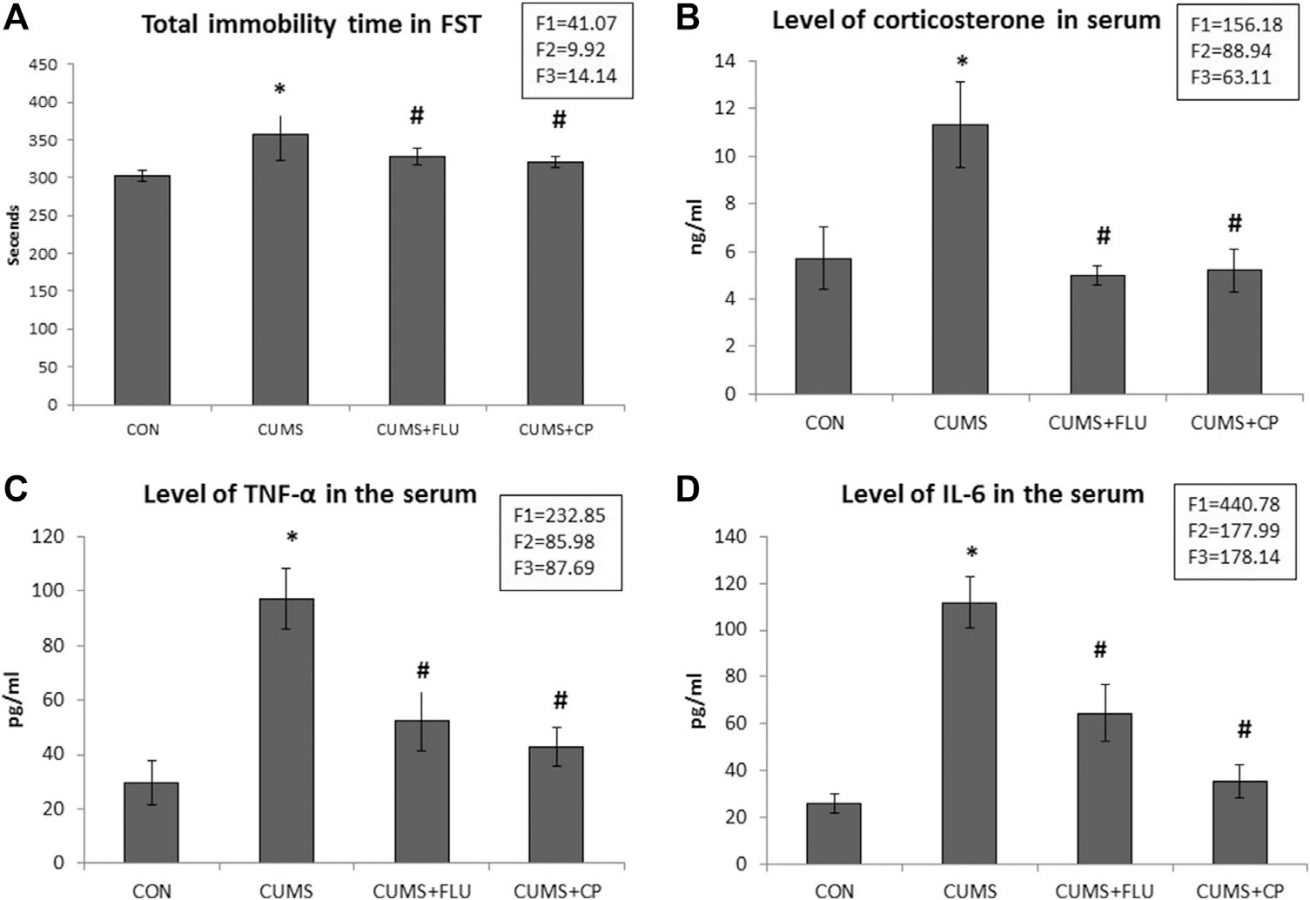
Immobility time during the forced swimming test (FST) **(A)**, serum corticosterone level **(B)**, TNF-α serum level **(C)**, and IL-6 serum level **(D)** in the studied groups. Data are analyzed using mixed-model two-way ANOVA based on the Bonferroni post hoc test. Results are expressed as mean ± SD (*n* = 10), dF = 36, F1 = effect of stress, F2 = effect of treatment, F3 = interaction between stress and treatment. A *p* value <0.05 was considered significant. * significance versus the control group (CON), # significance versus the chronic unpredictable mild stress (CUMS) group. CON: control, CUMS: chronic unpredictable mild stress, Flu: fluoxetine, CP: *Cucurbita pepo*.

The immobility time during the FST was significantly higher (*p* = 0.03) in the CUMS group than the control, whereas it was significantly lower (*p* = 0.01, *p* = 0.001) in FLU- and CP-treated groups than in the CUMS group (Figure 1A).

### Results of CP Extract Analysis Using GC-MS

**FIGURE 2:**
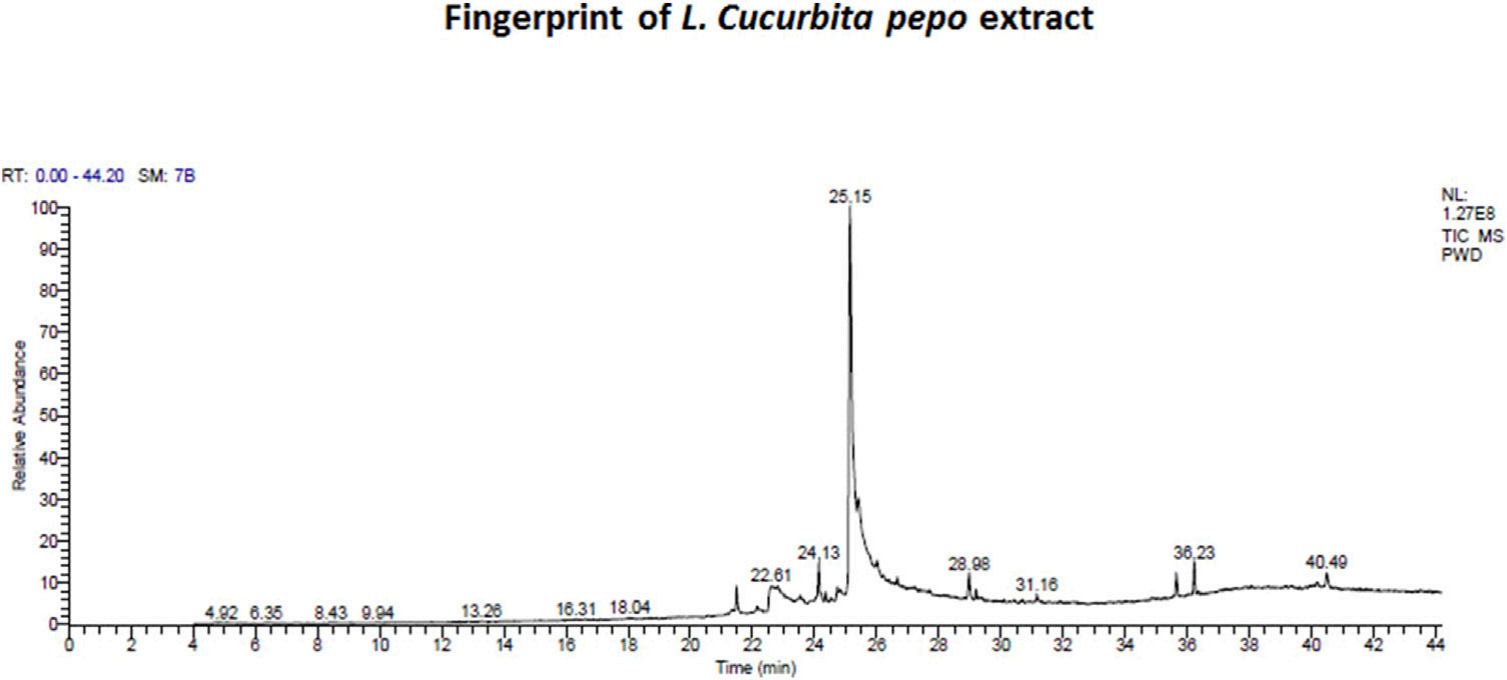
Fingerprint of chromatogram of *L. Cucurbita pepo* extract prepared by using a trace gas chromatography mass spectrometer.

The main compounds detected in CP mainly include oleic acid (about 56%), palmitic acid (about 8.9%), linolenic acid 3.5%, and linoleic acid 2.8% besides many other compounds (Table 2; Figure 2). Among the compounds of CP that were reported to have anti-inflammatory effect, there are oleic acid, palmitic acid, linolenic acid, betulin, and linoleic acid, while those with an antioxidant effect are palmitic acid and 10-octadecenoic acid and methyl ester.

**TABLE 2:** Components of *Cucurbita pepo* L. extract identified using gas chromatography and mass spectrometer (GC–MS) analysis.

SN	Name of the compound	Molecular formula	Molecular weight	Retention time (minutes)	Relative percentage	NIST match factor (Mikaia et al., 2014)	Activity
1.	Hexadecanoic acid, methyl ester (palmitic acid methyl ester)	C_17_H_34_O_2_	270	21.50	3.02	839	Anti-inflammatory action through inhibition of the cyclooxygenase II enzyme (Hema et al., 2011)Antioxidant, hypocholesterolemic, lubricant, antiandrogenic (Duke, 1992)
2.	Hexadecanoic acid (palmitic acid)	C_16_H_32_O_2_	256	22.61	8.90	821	Anti-inflammatory through inhibition of phospholipase A2 (Aparna et al., 2012) Antioxidant, hypocholesterolemic, lubricant, antiandrogenic (Duke, 1992)
3.	9-octadecenoic acid (Z)-	C_18_H_34_O_2_	256	22.82	4.40	894	-
4.	10-octadecenoic acid, methyl ester	C_19_H_36_O_2_	296	24.13	4.82	804	Antibacterial, antifungal, antioxidant (Asghar and Choudahry, 2011)
5.	Butyl 9,12,15-octadecatrienoate	C_22_H_38_O_2_	334	24.73	1.60	743	No activity reported
6.	9-octadecenoic acid (oleic acid)	C_18_H_34_O_2_	282	25.14	56.59	901	Anti-inflammatory actions through peroxisome proliferator-activated receptor gamma (PPAR-γ) activation (Song et al., 2019)
7.	9,12-octadecadienoic acid (Z,Z)- (linolenic acid)	C_18_H_32_O_2_	280	25.42	3.52	855	Anti-inflammatory, hypocholesterolemic, antiandrogenic, antihistaminic, antieczemic (Duke 1992)
8.	*cis*-13-octadecenoic acid (linoleic acid)	C_18_H_34_O_2_	282	26.01	1.75	796	Anti-inflammatory, antiandrogenic, antileukotriene-D4, hypocholesterolemic, flavor (Duke 1992)
9.	9-octadecenoic acid (Z)-, anhydride (oleic anhydride)	C_36_H_66_O_3_	546	28.98	3.60	800	Anti-inflammatory (Song et al., 2019)
10.	Linoleic acid ethyl ester (ethyl linoleate)	C_20_H_36_O_2_	308	29.21	1.08	782	No activity reported
11.	Stigmast-5-en-3-Ol	C_29_H_50_O	414	35.65	3.08	725	Decrease endothelial leukocyte and platelet adhesion (Duke, 1992; Kikuchi et al., 2015)
12.	Betulin	C_30_H_50_O_2_	442	40.49	2.35	644	Anti-inflammatory and antitumor effect (Kikuchi et al., 2015; Jonnalagadda et al., 2017)

### Biochemical Assessment

#### Corticosterone Level in the Serum

The corticosterone level significantly increased (*p* < 0.001) in untreated rats exposed to CUMS compared to that in the control rats, while those treated with FLU and CP showed a significant reduction (*p* < 0.001) in serum corticosterone level compared to that in the untreated rats exposed to CUMS (Figure 1B).

### TNF-α and IL-6 Levels in the Serum

At the end of the experiment, assessment of serum TNF-α and IL-6 showed that they were significantly elevated (*p* < 0.001) in the CUMS group, whereas they showed a significant reduction (*p* < 0.001) in FLU- and CP-treated groups compared with those in the untreated rats exposed to CUMS (Figures 1C,D).

### MDA, SOD, GPX, and CAT Levels in the Serum

**FIGURE 3:**
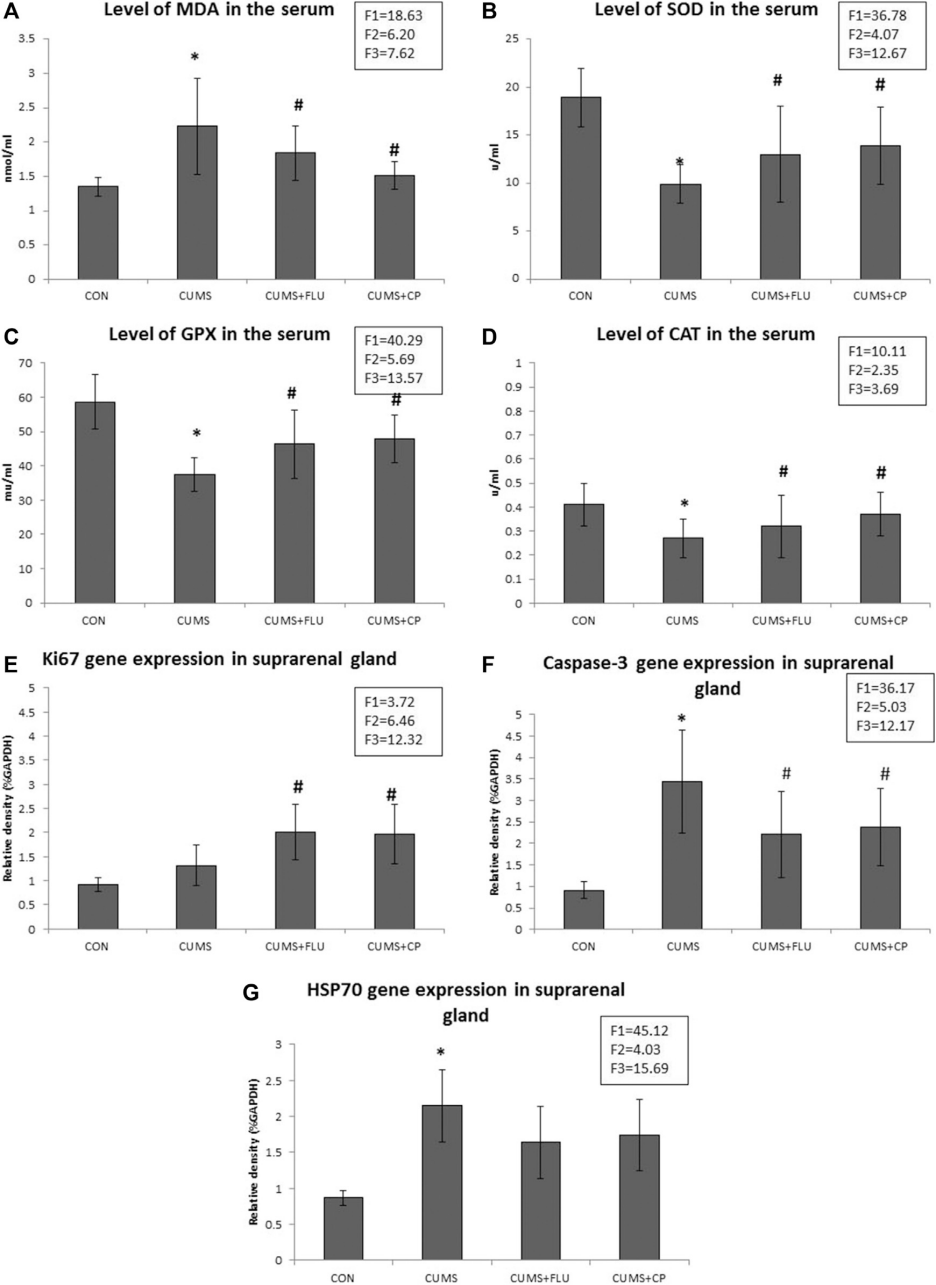
Levels of MDA **(A)**, SOD **(B)**, GPX **(C),** and CAT **(D)** assessed in the serum of the studied groups using ELIZA. Levels of gene expression of Ki67 **(E)**, caspase-3 **(F),** and HSP70 **(G)** assessed in the suprarenal glands of the studied groups using qRT-PCR. Data are analyzed using mixed-model two-way ANOVA based on the Bonferroni post hoc test. Results are expressed as mean ± SD (*n* = 10), dF = 36, F1 = effect of stress, F2 = effect of treatment, F3 = interaction between stress and treatment. A *p* value <0.05 was considered significant. * significance versus the control group (CON), # significance versus the chronic unpredictable mild stress (CUMS) group. CON: control, CUMS: chronic unpredictable mild stress, Flu: fluoxetine, CP: *Cucurbita pepo*.

It was noticed that the level of serum MDA was significantly higher (*p* < 0.001) in the CUMS group than in the control rats, while that in CP-treated groups was significantly lower (*p* = 0.003) than that in untreated rats exposed to CUMS. FLU administration could not significantly reduce the MDA level in rats exposed to CUMS (Figure 3A).

In contrast, levels of SOD, GPX, and CAT in the serum of the CUMS group were significantly lower (*p* < 0.001, *p* < 0.001, *p* = 0.002) than those in the serum of the control group, while their levels showed a significant increase (*p* = 0.01, *p* = 0.003, *p* = 0.03) in the CP-treated group compared to those in the CUMS-exposed group. FLU administration could not significantly increase SOD, GPX, and CAT in rats exposed to CUMS (Figures 3B–D).

### Gene Expression of Ki67, Caspase-3, and HSP70

RT-PCR revealed insignificantly high Ki67 gene expression (*p* = 0.14) in the adrenal glands of the CUMS group compared to the control group, while that of FLU- and CP-treated groups showed significant high expression (*p* = 0.02, *p* = 0.01) compared to that of the CUMS-exposed group (Figure 3E).

Regarding caspase-3, its gene expression was significantly increased (*p* < 0.001) in the adrenal glands of the CUMS group compared to the control group, while that of FLU- and CP-treated groups showed a significant decrease (*p* = 0.03, *p* = 0.04) compared to that of the CUMS-exposed group (Figure 3F).

When it came to HSP70 gene expression, it recorded a significant higher (*p* < 0.001) levels in the adrenal glands of the CUMS group than in the control group, while that of FLU- and CP-treated groups showed an insignificant decrease (*p* = 0.07, *p* = 0.08) compared to that of the CUMS-exposed group (Figure 3G).

### Histopathological Assessment

**FIGURE 4:**
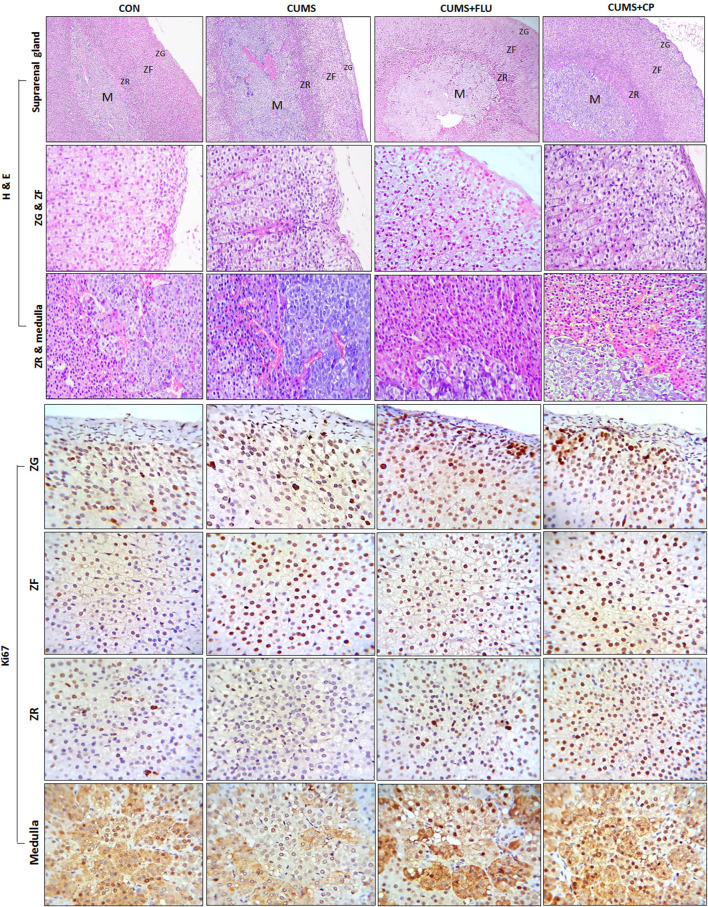
Sections of the adrenal gland of studied groups stained with H&E staining and Ki67 antibody (H&E x100 and 40, immunohistochemical staining X100). ZG, Zona glomerulosa, ZF: Zona fasciculata, ZR: Zona reticularis. CON: control, CUMS: chronic unpredictable mild stress, Flu: fluoxetine, CP: *Cucurbita pepo*.

Adrenal glands of control rats have an intact structure with a preserved architecture of the cortex included zona glomerulosa, zona fasciculata, and zona reticularis as well as the medulla (Figure 4). Adrenal glands of CUMS-exposed rats showed many cells in the ZG, ZF, and ZR with obvious vacuolation and degenerated nuclei and sometimes deeply stained nuclei with moderate thickening in the capsule and connective tissue trabeculae. Many cells of the ZR appeared dark with brown lipofuscin pigments. On the other hand, adrenal glands of CP-treated rats showed fewer obviously vacuolated cells and deeply stained nuclei in the ZG, and ZF and ZR revealed few dark cells (Figure 4).

Morphometric measurements showed that the thickness of the ZG and ZR showed an insignificant increase after exposure to CUMS, while they were significantly increased in FLU- (*p* = 0.01, *p* = 0.02) and CP- treated groups (*p* = 0.02, *p* = 0.001), respectively. Regarding the thickness of the ZF, it was significantly increased (*p* = 0.02) in the CUMS-exposed group, and it was further increased, but insignificantly, in FLU- and CP-treated groups compared to that in the untreated CUMS group.

**FIGURE 5:**
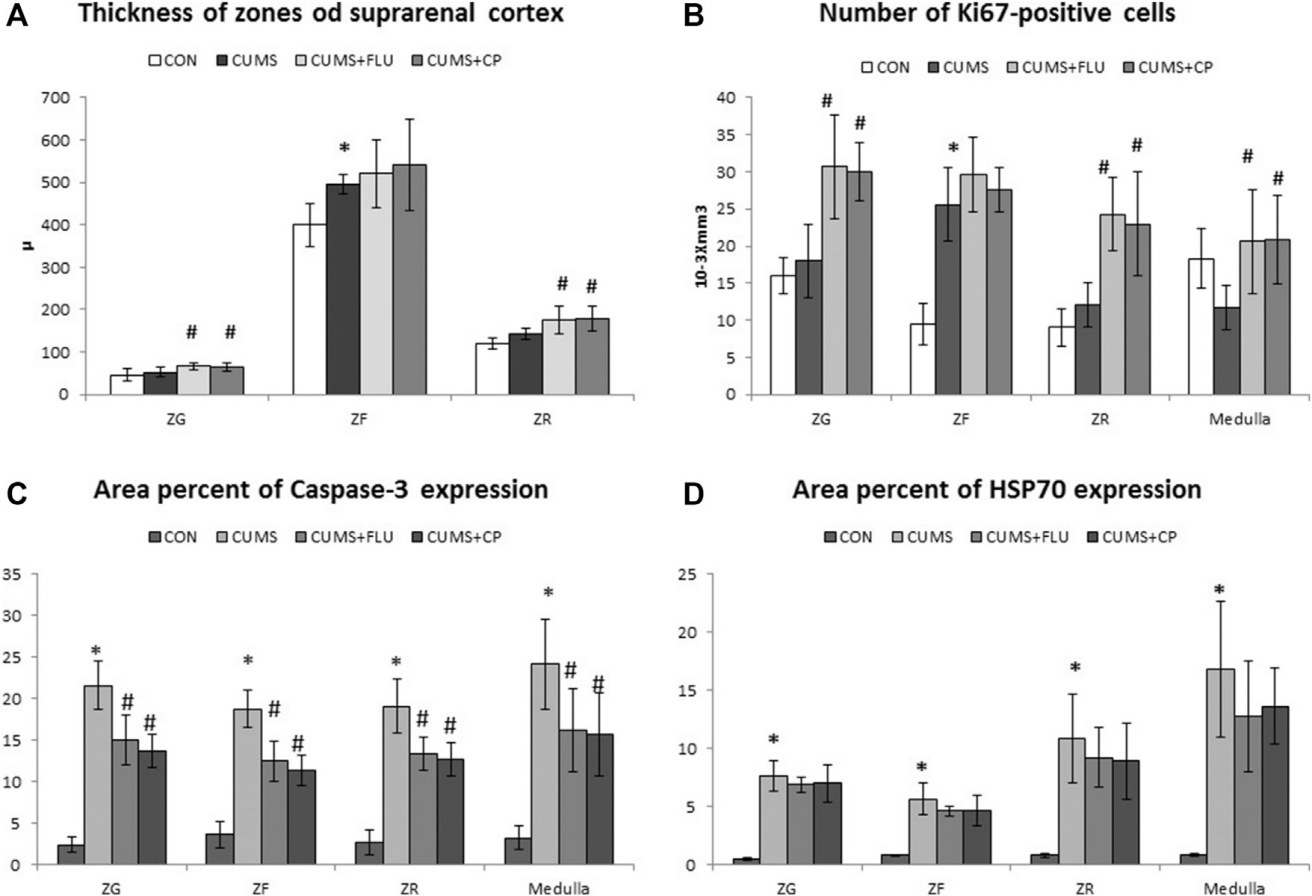
Thickness of the suprarenal cortical zones **(A)**, Immuno-expression of Ki67 **(B)**, caspase-3 **(C)**, and HSP70 **(D)** in the studied groups. Data are analyzed using mixed-model two-way ANOVA based on the Bonferroni post hoc test. Results are expressed as mean ± SD (*n* = 10), dF = 36. Results are expressed as mean ± SD (*n* = 10). A *p* value <0.05 was considered significant. * significance versus the control group (CON), # significance versus the chronic unpredictable mild stress (CUMS) group. ZG: zona glomerulosa, ZF: zona fasciculata, ZR: zona reticularis. CON: control, CUMS: chronic unpredictable mild stress, Flu: fluoxetine, CP: *Cucurbita pepo*.

In order to determine whether cellular hyperplasia was restricted to a specific subregion of the adrenal gland, immunohistochemical labeling for Ki67 (the marker of the dividing cell) was used. The number of Ki67-positive proliferating cells was significantly increased (*p* < 0.001) in the ZF after exposure to CUMS, while treatment with FLU and CP insignificantly increased it compared to the untreated CUMS group. Although the number of proliferating cells was insignificantly increased in the ZG and ZR and insignificantly decreased in the medulla of the CUMS group compared to the control group, treatment with FLU and CP significantly increased the number of proliferating cells in the ZG (*p* = 0.001, *p* = 0.003), ZR (*p* < 0.001) and medulla (*p* = 0.004, *p* = 0.003) compared to the untreated CUMS group (Figures 4, 5).

**FIGURE 6:**
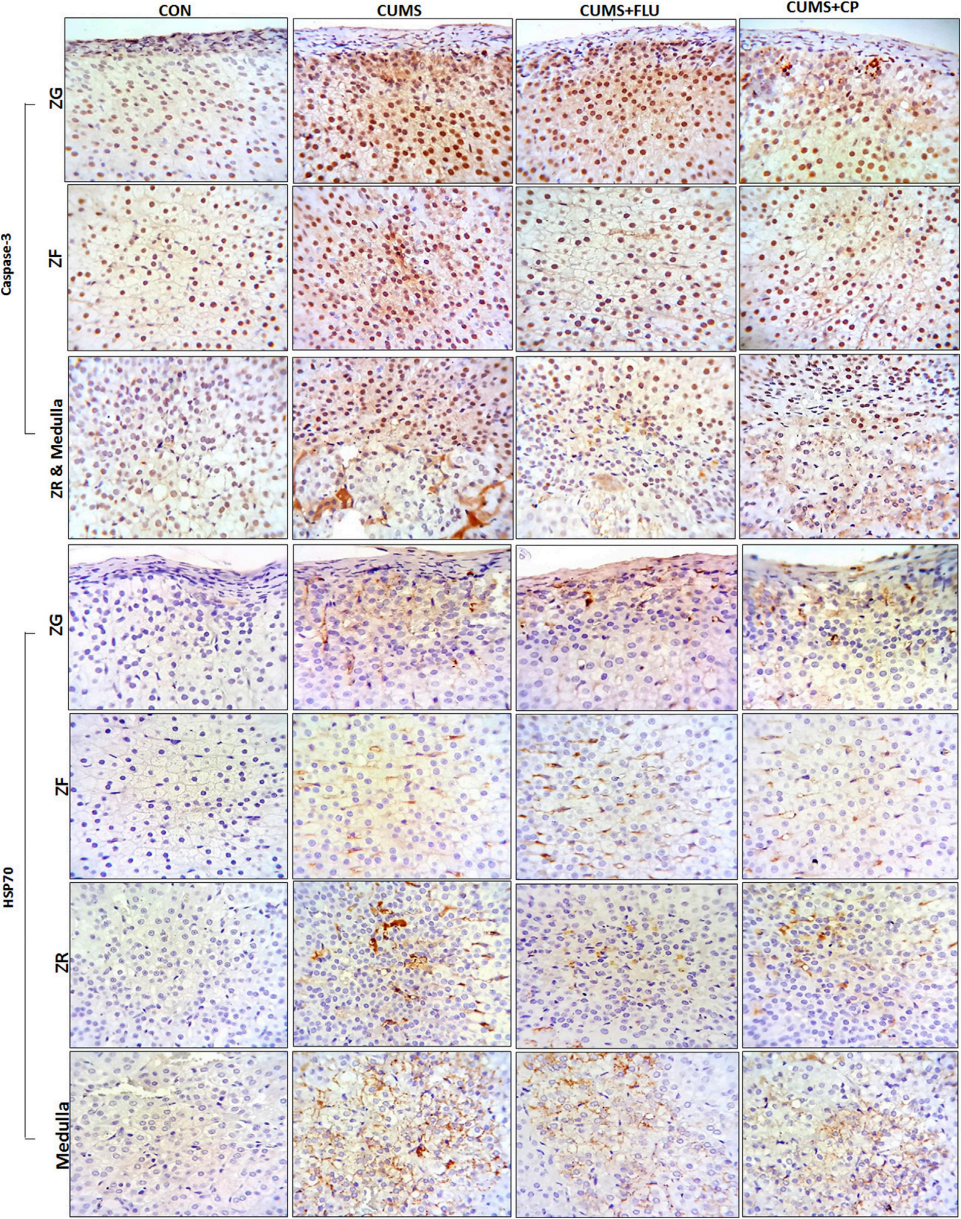
Sections of the adrenal gland of studied groups stained immunohistochemically with caspase-3 and HSP70 antibodies. ZG: zona glomerulosa, ZF: zona fasciculata, ZR: zona reticularis. CON: control, CUMS: chronic unpredictable mild stress, Flu: fluoxetine, CP: *Cucurbita pepo*.

Immunohistochemical staining using caspase-3 was performed to assess apoptosis (Figure 6). It was noticed that the percent area of caspase-3 expression significantly increased (*p* < 0.001) in all zones of the gland following exposure to CUMS for 4 weeks in comparison to that of the control group. The CP-treated group showed a significant reduction in caspase-3 expression in the ZG (*p* < 0.001), ZF (*p* < 0.001), ZR (*p* < 0.001, *p* = 0.005), and medulla (*p* = 0.004, *p* = 0.001), in comparison to that of the CUMS group (Figures 5, 6).

The area percent of HSP70 expression significantly increased (*p* < 0.001) in all zones of the cortex as well as in the medulla of the adrenal gland of the CUMS group compared to that of the control group, whereas it was insignificantly reduced in all zones and medulla of FLU- and CP-treated groups compared to that of the CUMS group (Figures 5, 6).

## Discussion

Modern society is obviously full of stress that may arise from daily problems, health care, and job-related issues. These stressors may cause the so-called stress-related disorders (Clayton and Mccance, 2014). In this study, the ethanolic extract of the flesh and peel of *Cucurbita pepo* L. was used to investigate its potential in alleviating the CUMS-induced depressive behavior and its impact on the adrenal gland. This was proved by a significant increase in the serum corticosterone level and verified by a prolonged immobility time during the FST. These findings were consistent with those previously reported (Tang et al., 2015).

Both TNF-α and IL-6 were reported to be involved in pathogenesis of depression (Taraz et al., 2015; Pedraz-Petrozzi et al., 2020). It was reported that mice deficient in IL-6 or TNF-α receptors are resistant to depressive behaviors (Viana et al., 2010). Therefore, they were assessed in this study, and it was observed that both TNF-α and IL-6 were significantly increased in the serum of rats exposed to CUMS for 4 weeks, indicating that they were depressed. This finding was supported by Numakawa et al. (2014) in mice with behavioral despair and by Taraz et al. (2015) in patients with depression.

In this study, administration of pumpkin extract was associated with amelioration of the depressive behavior, evident by the significant reduction of the immobility time of the FST and confirmed by the significant reduction in corticosterone and inflammatory cytokines TNF-α and IL-6 in the serum. These findings were supported by Kim Nr et al. (2016) who reported that SSP for 28 days decreased the level of inflammatory cytokines in the depressed rats. The anti-inflammatory activity of pumpkin might be attributed to the active compounds of the pumpkin such as oleic and palmitic acids and estradiol.

Downregulation of oleic acid, palmitic acid, and linoleic acid was described in depression (Conklin et al., 2010; Martín et al., 2010). It was reported that oleic acid–mediated neuroprotection is linked to its anti-inflammatory effect mediated by peroxisome proliferator–activated receptor gamma (PPAR-γ) activation (Song et al., 2019). Adding to that, palmitic acid was reported to inhibit phospholipase A(2), and therefore considered as an anti-inflammatory compound (Aparna et al., 2012). Estradiol was one of the compounds of the pumpkin detected in this study. Estradiol was reported to induce an anti-inflammatory effect and subsequently alleviate the depressive behavior (Xu et al., 2015). Another mechanism by which estradiol can induce an antidepressant effect is upregulation of expression of brain-derived neurotropic factor (BDNF) and ERK phosphorylation by the activation of ER-β in the brain (Yang et al., 2010).

In this study, the serum level of MDA was increased, while SOD, GPX, and CAT levels were decreased in the CUMS-exposed rats. Similar changes were documented following the exposure to CUMS in previous studies, and they added that these biochemical changes resulted in oxidative stress and impairment of HPA (Nabavi et al., 2015). Liu T. et al. (2015) also reported that development of depression may be attributed to the low content of antioxidants in the body. The antioxidant properties of the pumpkin fruit extract were frequently reported (Bahramsoltani et al., 2017). In addition, the flesh and peel of the *Cucurbita pepo* L. was reported to process higher antioxidant activity than the other parts, for example, seeds (Oyeleke et al., 2019). Pumpkin extract administration, in many previous studies, significantly increased the levels of SOD and GPX and reduced MDA in the serum of mice (Guo-Hua et al., 2000; Dang, 2004), and this effect was also documented in this study. Therefore, the antioxidant activity of pumpkins could be behind its antidepressant effect evident in this study. This is in a line with the observations of Kim Nr et al. (2016). It was reported that natural products with anti-inflammatory, antioxidant, and anti-fatigue effects also have an antidepressant-like effect (Jeong et al., 2015). All these previous activities were proved in pumpkins.

Increased cytoplasmic vacuolation of adrenal cortical cells, observed in the CUMS group in this study, might be attributed to increase demand for lipids, which forms the cornerstone for cortisol synthesis. Cigankova et al. (2005) observed that long-lasting experimental hypodynamia resulted in coalescence of multiple cytoplasmic lipid droplets of cortical cells. These lipid droplets contain cholesterol, which is the principal precursor in the synthesis of steroid hormones. However, Koldysheva and Lushnikova (2008) reported that stress resulted in a significant decrease in lipid droplets in cells of the adrenal cortex, especially ZF (Koldysheva and Lushnikova, 2008). It was described that upon exposure to physical stress, pain stimuli are transmitted to the hypothalamus, resulting in CRH secretion into the hypophyseal portal system, which increases ACTH secretion and stimulates its receptors in the ZF and ZR to increase cortisol secretion (Patti et al., 2018). The thickened adrenal capsule and trabeculae observed in CUMS-exposed rats were previously reported (El-Desouki et al., 2011; Altayeb and Salem, 2017) as a result of increased collagen synthesis by fibroblasts on exposure to immobilization stress.

Many cells in the adrenal glands of CUMS-exposed rats showed deeply stained nuclei, indicating that they underwent apoptosis that was confirmed by caspase-3 immunohistochemical staining. Similar observation was reported by Altayeb and Salem, (2017), while they were studying the effect of immobilization stress on adrenal glands of rats. Liu Q. et al. (2015) reported that excessive oxidative stress, evident following exposure to CUMS in this study, modifies the expression levels of “apoptosis-related genes” and induces cell apoptosis and degeneration through signaling pathways of Bcl-2, Bax, and caspase-3. This mechanism was confirmed in this study by RT-PCR that revealed significant upregulation of caspase-3 gene expression. On reviewing the literature, there was no direct effect of *Cucurbita pepo* on caspase-3–mediated apoptosis in different tissues. It seems that reduction in caspase-3–positive apoptotic cells detected in the adrenal glands was attributed to the antioxidant effect of *Cucurbita pepo* documented in this study. This is supported by many previous studies conducted on other plants and herbs with significant antioxidant activities (Banagozar Mohammadi et al., 2019; Ghazizadeh et al., 2020).

This study showed that exposure to CUMS increased Ki67-positive cells in the adrenal cortex that was significantly evident in the ZF, indicating an increased number of proliferating cells, and as a result, the thickness of this layer was significantly increased. On the other hand, the number of Ki67-positive proliferating cells insignificantly decreased in the medulla in response to CUMS. These observations were in a line with those of Ulrich-Lai et al. (2006) who reported an increase in Ki67-positive cell nuclei only in the outer ZF after exposure to chronic variable stress, while the medulla showed cellular hypertrophy and not hyperplasia. Moreover, Laborie et al., (2003) reported a generalized increase in medullary function after exposure to chronic stress, suggesting that chronic stress may lead to medullary hypertrophy. In this study, administration of the pumpkin extract significantly increased cell proliferation, evident by the upregulation of the Ki67 gene and immuno-expression in almost all zones and the medulla of the adrenal gland compared to CUMS. This explained increased thickness of the gland zones recorded in this study. It also may compensate the apoptotic effect induced by exposure to CUMS. This finding is supported by Kim Hy et al. (2016) who reported a significant upregulation of the mRNA expression of Ki67 and proliferation of splenocytes isolated from the spleen of BALB/c mice treated with SSP, streamed and *Cucurbita moschata Duch* and its major component, β-carotene.

In the present study, exposure to CUMS significantly increased gene expression and immuno-expression of HSP70 in all zones of the adrenal gland. In concordance with that, Zheng et al. (2008) also proved that augmented HSP70 expression after exposure to stressful conditions modulates inflammatory responses by inhibiting activation of the inflammatory transcription factor, the nuclear factor-kappa B (NF-kappaB). In addition, HSP70 may directly interfere with apoptosis and necrosis (Yenari et al., 2005). More recently, Li et al. (2019) reported an upregulation of HSP70 expression in the porcine adrenal gland tissue following exposure to heat stress, which indicates the role of HSP70 in adrenal gland injury and emphasizes its relevance to inflammatory responses. The rapid induction of HSP70 in response to stress is considered essential to the cellular protection process.

**FIGURE 7:**
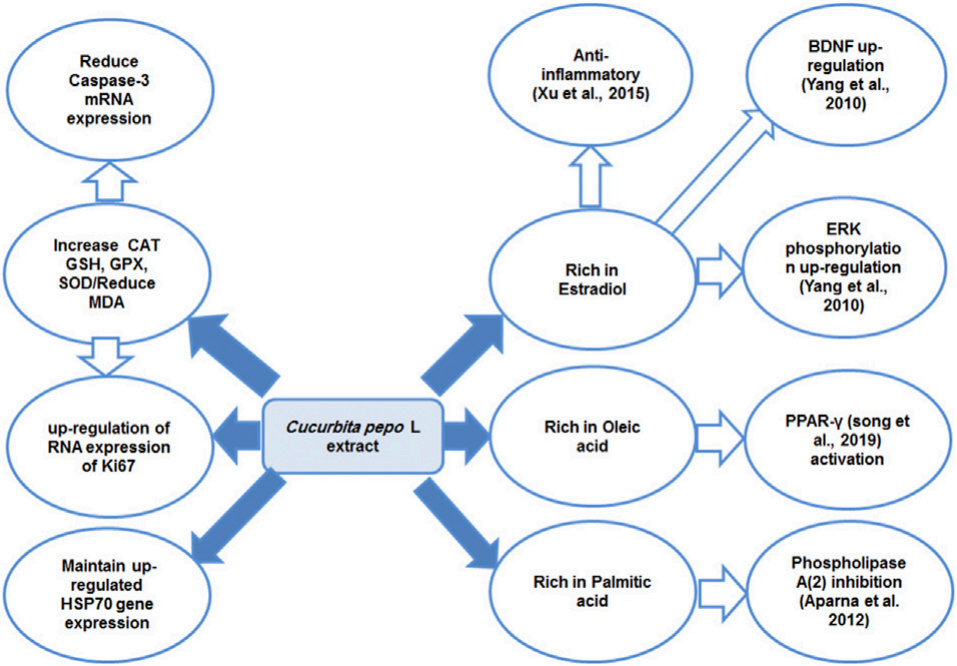
Diagram summarizing the proposed mechanism of antidepressant effect of the *Cucurbita pepo* L extract. Peroxisome proliferator–activated receptor gamma (PPAR-γ), extracellular signal–regulated kinase (ERK), brain-derived neurotropic factor (BDNF), catalase (CAT), glutathione peroxidase (GPX), reduced glutathione (GSH), and superoxide dismutase heat shock protein (HSP70).

In our study, treating CUMS-exposed rats with pumpkins was associated with an insignificant reduction in HSP70 expression which was associated with reduced apoptosis and inflammatory responses. This might point to the beneficial effect of the relatively high level of HSP70 induced by both FLU and CP as it resulted in the reduction of caspase-3–mediated apoptosis and release of inflammatory cytokines. Although the effect of pumpkins on heat shock protein family was not previously described, oleic acid, which represents the main constituents of pumpkins used in this study, was described to downregulate the expression of the HSP60 in the human T lymphocyte cell line (Martins De Lima et al., 2004), which is supportive to our study finding. The proposed mechanism of action of *Cucurbita pepo* as an antidepressant-like substance is summarized in Figure 7. Among the limitations of this study was the inability to further investigate the in-depth mechanism of the antidepressant effect of CP, and therefore, further study is encouraged to do that.

In conclusion, this study provides science-based evidence of the efficacy of *Cucurbita pepo* L. extract to alleviate CUMS-induced behavioral and biochemical changes as well as the histopathological impact on the adrenal glands. These effects were evident through the downregulation of apoptosis and HSP70 expression and seemed to be mediated through the antioxidant and anti-inflammatory effect of the *Cucurbita pepo* L. extract. Although this study and some other recent studies have documented the antidepressant-like effect of *Cucurbita pepo* and explored its mechanism, further studies including clinical studies are needed to affirm this effect in humans.

## Data Availability

The original contributions presented in the study are included in the article/Supplementary Material, further inquiries can be directed to the corresponding authors.
